# A Case of Extrauterine Endometrial Stromal Sarcoma in the Colon Diagnosed Three Decades after Hysterectomy for Benign Disease

**DOI:** 10.1155/2013/202458

**Published:** 2013-04-24

**Authors:** Andres Ayuso, Oluwole Fadare, Dineo Khabele

**Affiliations:** ^1^Department of Obstetrics and Gynecology, Vanderbilt University School of Medicine, Nashville, TN 37232, USA; ^2^Department of Pathology, Microbiology and Immunology, Vanderbilt University School of Medicine, Nashville, TN 37232, USA; ^3^Division of Gynecologic Oncology, Department of Obstetrics and Gynecology, Vanderbilt University School of Medicine, B1100 Medical Center North, Nashville, TN 37232, USA

## Abstract

Extrauterine endometrial stromal sarcoma (ESS) is rare and typified by delayed recurrence of primary ESS. Here, we report an unusual case of colonic ESS in a woman with a remote history of hysterectomy. An 80-year-old woman, with a history of hysterectomy and bilateral salpingo-oophorectomy for abnormal bleeding and endometriosis 37 years prior to presentation, was diagnosed with ESS in the colon. She was treated with laparoscopic low anterior resection, followed by megestrol acetate, and has been in remission for more than 4 years. This case highlights the rarity of extra-uterine ESS in the colon, especially in the absence of a known history of primary uterine ESS. The patient's history of endometriosis may have been a predisposing risk factor. ESS in the colon may be treated successfully with surgical resection and progestin therapy. Indefinite surveillance is recommended to monitor for late recurrences.

## 1. Introduction 

Endometrial stromal sarcoma (ESS) tumors account for approximately 0.2% of all uterine malignancies [1]. These tumors resemble endometrial stromal cells in the proliferative stage and are often of low grade, slow growing, and indolent. However, approximately 50% of women are diagnosed with recurrent disease, sometimes decades after the initial diagnosis. The most common sites of extra-uterine ESS are in the pelvis. Gastrointestinal involvement is rare. Here, we report an unusual case of colonic ESS in a woman with no documented prior history of primary uterine ESS, diagnosed more than three decades after hysterectomy.

## 2. Case Presentation 

**Figure 1:**
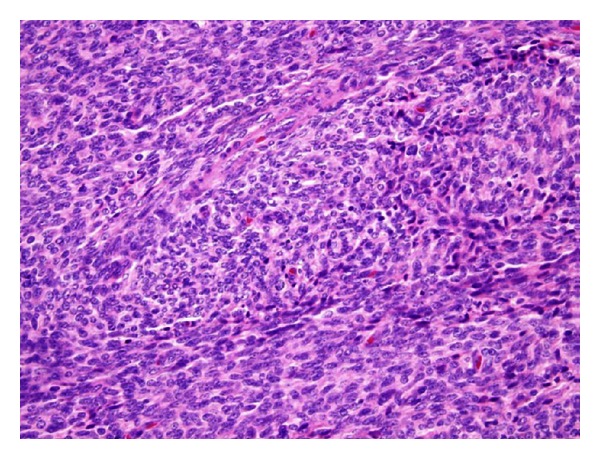
A representative hematoxylin and eosin stain of the colonic ESS. The tumor is comprised of round to oval plump spindle-shaped nuclei with minimal nuclear atypia and rare mitotic features. Small, prominent arterioles are interspersed between the stromal cells.

An 80-year-old woman, gravida 3, para 2, with a history of total abdominal hysterectomy and bilateral salpingo-oophorectomy for abnormal bleeding and endometriosis performed 37 years prior, presented to the hospital with bright red rectal bleeding and chronic rectal discharge. She denied vaginal bleeding or discharge. She had been prescribed raloxifene for osteoporosis prevention and intermittent treatment with vaginal conjugated estrogen cream for urogenital atrophy, but had not seen a gynecologist since her hysterectomy. Colonoscopy and biopsy were negative for malignancy. Further evaluation with computed tomography (CT) imaging demonstrated a 5 cm soft tissue mass in the pelvis involving the sigmoid colon. She underwent a laparoscopic-assisted low anterior colon resection and biopsy of left pelvic sidewall and omental nodules. The sigmoid colon mass revealed a low-grade endometrial stromal sarcoma, involving the mucosa, muscularis, and adjacent peritoneal tissue, including three pericolonic soft tissue nodules and the left pelvic sidewall nodule. The proximal and distal margins, 18 regional lymph nodes, and omental biopsy were negative for malignancy. The tumor showed the characteristic morphology of a low-grade spindle neoplasm with uniform proliferation of small round to oval cells, minimal nuclear atypia or mitoses, and small nucleoli (Figure 1). Small, prominent arterioles were scattered among the stromal cells. There was no evidence of necrosis. The tumor cells stained positive for CD10, panCK, EMA, and desmin (focal). No residual uterine stump or ovaries were identified intraoperatively or on the final histopathological evaluation. Additional metastatic workup was negative. 

The patient was treated with megestrol acetate 80 mg by mouth twice a day, decreased to 40 mg daily and eventually discontinued after 3 years, due to the patient's inability to tolerate the side effects. The patient has been followed closely with examination, CT imaging and endoscopy, and she has remained without evidence of recurrent ESS for over 4 years. 

## 3. Discussion

This case is one of only two reported cases of extra-uterine low-grade ESS diagnosed more than 30 years after hysterectomy for benign disease. The other case was a patient with a remote history of hysterectomy for leiomyoma 38 years prior to the diagnosis of low-grade ESS in the small bowel [2]. The etiology of this patient's extra-uterine ESS is not entirely clear. While it would be unusual for this patient to have been misdiagnosed with primary uterine ESS more than 30 years prior, this possibility is a consideration due to previous reports of remote recurrences up to two decades after the primary diagnosis. Unfortunately, slides from this patient's hysterectomy were not available for comparison. 

 ESS of the colon is exceedingly rare, with only 7 reported cases in the literature [3–8]. Documented endometriosis was associated with 6 of 7 (86%) previously reported cases. A review of multiple case series shows that extra-uterine ESS is associated with foci of endometriosis in the peritoneal cavity [2, 9–11]. Although this patient reported a history of endometriosis, there was no gross or histological evidence of endometriosis at the time of colonic resection. 

Primary extra-uterine ESS is more common in premenopausal women [2, 9–11], suggesting a hormonal influence. Since the patient had been prescribed vaginal conjugated estrogen cream for urogenital atrophy and raloxifene for osteoporosis, another potential contributing factor to the development of the colonic ESS in this patient is hormonal therapy. The systemic absorption of vaginal conjugated estrogens is well known [12] and theoretically may have contributed to the development of ESS. While there is one reported case of a malignant mixed mesodermal tumor diagnosed in a patient taking raloxifene for osteoporosis [13], in general, raloxifene is not known to be associated with endometrial malignancies [14]. Thus, raloxifene is not likely to have had an effect. 

In summary, we report a rare case of extra-uterine ESS in the colon diagnosed 37 years after hysterectomy reportedly for abnormal bleeding and endometriosis. Endometriosis was not confirmed histologically at the time of this patient's colonic resection. However, based on the strong association with extra-uterine ESS and endometriosis observed in the literature this patient's tumor may have arisen from transformation of pelvic peritoneal endometriosis. We acknowledge that the unavailability of the pathological slides from the previous hysterectomy and lack of endometriosis on the colonic resection are the biggest limitations in making this assessment. Nevertheless, the possibility of ESS should be considered in the diagnosis of solid stromal tumors in the gastrointestinal tract in women with a history of endometriosis since the majority of cases in the literature report this association. Management with surgical resection is recommended, and adjuvant progestin therapy is reasonable. As in this case, long-term remissions can be achieved. However, continuous followup is recommended because of the risk of delayed recurrence.
